# Mining-impacted rice paddies select for Archaeal methylators and reveal a putative (Archaeal) regulator of mercury methylation

**DOI:** 10.1038/s43705-023-00277-x

**Published:** 2023-07-15

**Authors:** Rui Zhang, Stéphane Aris-Brosou, Veronika Storck, Jiang Liu, Mahmoud A. Abdelhafiz, Xinbin Feng, Bo Meng, Alexandre J. Poulain

**Affiliations:** 1 Department of Biology, University of Ottawa, Ottawa, ON K1N 6N5 Canada; 2 Department of Mathematics and Statistics, University of Ottawa, Ottawa, ON K1N 6N5 Canada; 3 Department of Civil Engineering, Polytechnique Montréal, Montréal, QC H3C 3A7 Canada; 4 State Key Laboratory of Environmental Geochemistry, Institute of Geochemistry, Chinese Academy of Sciences, Guiyang, 550081 China; 5 University of Chinese Academy of Sciences, Beijing, 100049 China

**Keywords:** Environmental microbiology, Biogeochemistry

## Abstract

Methylmercury (MeHg) is a microbially produced neurotoxin derived from inorganic mercury (Hg), which accumulation in rice represents a major health concern to humans. However, the microbial control of MeHg dynamics in the environment remains elusive. Here, leveraging three rice paddy fields with distinct concentrations of Hg (Total Hg (THg): 0.21−513 mg kg^−1^ dry wt. soil; MeHg: 1.21−6.82 ng g^−1^ dry wt. soil), we resorted to metagenomics to determine the microbial determinants involved in MeHg production under contrasted contamination settings. We show that Hg methylating Archaea, along with methane-cycling genes, were enriched in severely contaminated paddy soils. Metagenome-resolved Genomes of novel putative Hg methylators belonging to *Nitrospinota* (*UBA7883*), with poorly resolved taxonomy despite high completeness, showed evidence of facultative anaerobic metabolism and adaptations to fluctuating redox potential. Furthermore, we found evidence of environmental filtering effects that influenced the phylogenies of not only *hgcA* genes under different THg concentrations, but also of two housekeeping genes, *rpoB* and *glnA*, highlighting the need for further experimental validation of whether THg drives the evolution of *hgcAB*. Finally, assessment of the genomic environment surrounding *hgcAB* suggests that this gene pair may be regulated by an archaeal toxin-antitoxin (TA) system, instead of the more frequently found *arsR*-like genes in bacterial methylators. This suggests the presence of distinct *hgcAB* regulation systems in bacteria and archaea. Our results support the emerging role of Archaea in MeHg cycling under mining-impacted environments and shed light on the differential control of the expression of genes involved in MeHg formation between Archaea and Bacteria.

## Introduction

Rice is an essential staple crop cultivated worldwide, roughly constituting 19% of the global calorie intake and occupying 10% of the global cropland [1, 2]. Rice consumption has been identified as a major source of human exposure to methylmercury (MeHg) [3, 4] that is a potent neurotoxin [5]. MeHg production is predominantly conducted by a group of microbes which genomes harbor two genes, *hgcA* and *hgcB*, coding for a putative methyltransferase and a ferredoxin, respectively [6, 7]. This gene pair exists in diverse microbial lineages, inhabiting a broad range of anoxic and hypoxic environments, suggesting extensive Hg methylation potential across the globe [8–10]. Considering the global biogeochemical cycling of Hg [11], and the prevalence of *hgcAB* genes, MeHg formation in rice paddies represents a worldwide issue affecting human health. Therefore, reducing the accumulation of MeHg in the environment, and thus human exposure to this contaminant, requires a comprehensive understanding of the various variables influencing the fluctuation of environmental MeHg concentrations (i.e., MeHg dynamics). One essential aspect that would contribute to our understanding of MeHg dynamics is to unravel the role of microbes in Hg transformation under diverse physiochemical constraints [12]. However, in rice paddy systems, particularly those impacted by mining activities releasing Hg-bearing residues and where Hg transformations occur, knowledge gaps remain regarding the microbial constraints on MeHg production.

Several studies have examined the distribution (i.e., diversity and relative abundance) of Hg-methylating microbes (i.e., using *hgcA* as a proxy) and their co-inhabiting microbial communities in Hg-impacted rice paddies due to mining activities [13–17]. This previous work has improved our understanding on how Hg influences the microbial community structure and how environmental variables affect the distribution of Hg methylators in such systems. However, rice paddies represent agricultural wetlands with extensive anthropic disturbances, such as the use of fertilizers and pesticides [18], straw amendments [19], and intermittent flooding and drying cycles [20]. These factors set rice paddies apart from other non-agricultural wetlands, and may contribute to higher Hg levels as well as substantial variation in microbial community structure, altogether leading to Hg buildup in rice [21]. Furthermore, an association between microbial methane and MeHg cycling is quickly emerging [22–24], as rice paddies are a non-negligible source of methane emission [25]. Yet, a thorough examination of the microbial functional potential is currently lacking for Hg-impacted rice paddies. Thus, a continued exploration of the rice paddy microbial and functional variations, especially the interplay between Hg and carbon cycling via methane production and degradation, considering the other geochemical parameters, is of practical significance in discerning the microbial constraints on MeHg accumulation.

Utilizing (meta)genomics and (meta)transcriptomics, previous studies attempted to explain MeHg dynamics using *hgc* gene abundance or expression levels (i.e., correlating MeHg concentration with the abundance of *hgc* genes and transcripts) [26–30]. However, it remains unclear whether *hgcA* abundance alone can reliably predict / correlate with MeHg levels. For instance, *hgcA* gene abundance was shown to poorly correlate with total Hg (THg) and/or MeHg concentrations in numerous environments the biogeochemical cycling of Hg, such as freshwater, wetland and organic-rich permafrost sediments [26, 27]. Inconsistent results have also been found in Hg-impacted rice paddies [15–17]. At the transcript level, incubation experiments conducted under sulfidogenic conditions using methylator *Desulfovibrio dechloroacetivorans* BerOc1 found no relationship between transcript abundance and net Hg methylation potentials [29]. Critically, our understanding of MeHg formation improves when coupling omics data with geochemical variables,. This has been demonstrated in two recent studies that showed 1) the synergistic effects of both *hgcA* abundance and soil DOM SUVA_254_ (i.e., as an indicator for Hg(II) bioavailability) explained the MeHg production in peat soil collected along a sulfate gradient [30]; and 2) the abundance or expression of *hgcA* and the concentration of dissolved Hg(II)-sulfide species collectively constrain Hg(II) methylation and MeHg accumulation in natural brackish water [28]. However, whether such relationships exist in Hg-impacted rice paddies is yet to be tested. An essential preliminary step is to estimate the abundance of *hgc* genes using improved bioinformatic methods, such as following a consensus protocol for *hgc* recovery [31] and employing hidden Markov models (HMMs) built with an updated collection of *hgc* sequences [32], which have not been done in previous rice paddy studies that have determined *hgc* abundance [15–17, 33].

The physiological role of Hg methylation represents another major knowledge gap. Several hypotheses have been formulated about the role of Hg methylation, including it being a metabolic accident that serves no selective advantage [34, 35], a detoxification mechanism [36], and an antimicrobial-producing process helping microbes compete in the primordial ocean [37]. Clearly, there is a need for additional evidence to explain the native role of Hg methylation, possibly through an evolutionary perspective [37, 38]. Furthermore, determining the regulatory system controlling the expression of *hgcAB* may inform on their functions in microbes. Recently, a study identified an *arsR* gene, co-transcribed with and upstream of *hgcAB* of several Hg methylators [39] It was later experimentally verified that a putative ArsR regulates the transcription of *hgcAB* and responds to arsenic, suggesting a link of Hg methylation to arsenic cycling [40].

Here, using marker-gene based and genome-resolved metagenomics, integrated with geochemical analysis, we investigated the community and functional composition of Hg methylators and their co-occurring microbiomes in mining-impacted rice paddies. Our sampling sites included a control site, an artisanal Hg mining site, and an abandoned Hg mining site, and represent paddy fields with unique contamination histories, which have resulted in distinct levels of Hg. In parallel, we tested whether THg exerted a selective pressure on *hgcA* by leveraging the contamination gradient using statistical phylogenetic analysis. Lastly, by pairing our metagenome-resolved genomes with the genomes of confirmed Hg methylators obtained from pure culture or environmental microbial isolates, we provided evidence showing that Hg methylators of bacterial and archaeal origin likely have different regulator genes controlling *hgcAB* expression—a finding that has a broader implication, beyond Hg-impacted rice paddies.

## Materials and methods

### Site and sampling

**Fig. 1:**
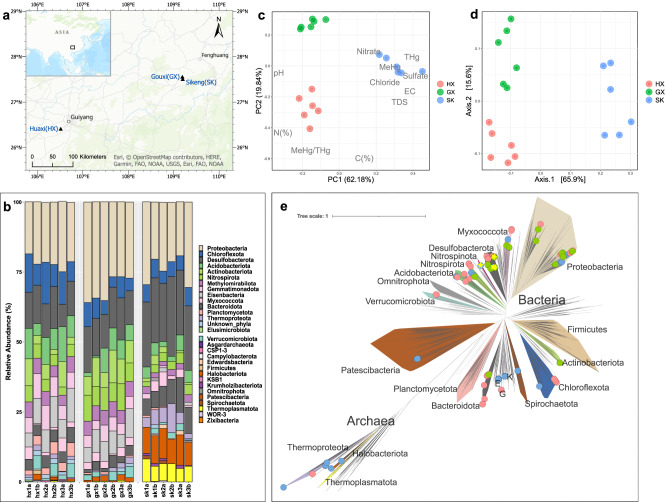
An overview of the microbial taxonomic diversity and soil geochemistry in the sampling sites. **a** Map of the sampling area showing the locations of the sites and their relative locations in Asia. **b** Relative abundance of bacteria and archaea at the phylum inferred using *rps11* gene coverage across the metagenomic assemblies. **c** A PCA biplot demonstrating the differences in paddy soil chemistry, plotted using data presented in Table S1. **d** PCoA of Bray-Curtis dissimilarity between microbial communities across the sampling sites at the phylum level, plotted using data shown in Table S2. **e** An unrooted phylogenomic tree of GTDB representative genomes and MAGs recovered in this study. MAGs from different rice paddies are assigned colored dots at the end of the tree branches (red: HX; green: GX; blue: SK). Hg methylator MAGs are indicated by the yellow star symbols. The tree includes 1368 representative genomes from GTDB and 159 MAGs retrieved from mining-impacted rice paddies. Major lineages (i.e., phylum), as well as lineages where our MAGs belong, are assigned colors arbitrarily. See Methods for details on genome inclusion and tree inference. (E Edwardsbacteria, K Krumholzibacteriota, G Gemmatimonadota).

Wanshan region in Guizhou province, Southwestern China, is known for its Hg mining and smelting, characterized by long-term, large-scale operations which have now ceased and short-term, small-scale artisanal Hg production, whose activities are poorly documented [41]. Such operations, depending on their duration and intensity, have led to local Hg contamination to various extents, which have created a gradient of Hg concentrations in the lands nearby, including rice paddies. Here it is such contemporary Hg gradients that we leveraged by targeting three sites with unique histories of mercury exposure (THg: 0.21−513 mg kg^−1^ dry wt. soil; MeHg: 1.21−6.82 ng g^−1^ dry wt. soil); Huaxi (denoted HX hereafter; 26°25′06.4′′N 106°30′50.6′′E), Gouxi (GX; 27°33′50.3′′N 109°11′29.5′′E), and Sikeng (SK; 27°30′50′′N 109°11′58′′E; Fig. 1a), as previously described [24]. We took six soil samples (1–20 cm below the soil-water interface) from each site and measured their physicochemical properties (Table S1). Soil sampling was conducted in August 2020, near the end of local rice cultivation period, where active Hg methylation had been observed [42]. Detailed descriptions of the mercury profiles across the sites can be found in Supplementary Text 1. Sample collection, preparation, paired-end metagenomics sequencing, and geochemical analyses can be found in Supplementary Text 2.

### Metagenomic assembly

Metagenomic short reads were trimmed using FastP (v0.20.1) [43] with default parameters to remove low-quality reads, adapters, and polyG sequences. Quality control checks of the trimmed reads were conducted using FastQC (v0.11.9) [44]. Trimmed reads from each sample were individually assembled into contigs with MEGAHIT (v1.2.9) [45]. Contigs were processed using Anvi’o (v7.1) for microbial taxonomic and functional diversity analyses [46]. Briefly, we used “anvi-script-reformat-fasta” to retain contigs with length greater than 1 kb from each sample for downstream analyses, and “anvi-gen-contigs-database” to calculate *k*-mer (*k* = 4) frequencies in contigs. Open reading frames (ORFs) were identified using Prodigal (v2.6.3), and prokaryotic single-copy core genes (SCGs) [47, 48] with “anvi-run-hmms” using HMMER (v3.2.1) [49]. To associate taxonomy information with the SCGs, we used “anvi-run-scg-taxonomy”, which searches the SCGs against the Genome Taxonomic Database (GTDB, release 202) using GTDB-tk (v1.5.0) [50, 51]. Functional annotation of the ORFs was conducted using “anvi-run-ncbi-cogs”, “anvi-run-kegg-kofams” and “anvi-run-pfams” against Clusters of Orthologous Groups released in 2020 (COG20) [52], Kyoto Encyclopedia of Genes and Genomes (KEGG) orthologs (or KOs) [53, 54] and Pfam (v35.0) [55], respectively. To calculate the coverage of the single-copy and the functional genes, we conducted metagenomic short read recruitment of the contigs greater than 1 kb using BWA MEM (v0.7.17) [56], and the subsequent SAM to BAM format conversion using samtools (v1.13) [57]. We sorted the BAM files using “anvi-init-bam” and profiled the sorted BAM files using “anvi-profile”.

We determined the abundance of SCGs in each sample with coverages as proxies using “anvi-estimate-scg-taxonomy”. The SCG with the highest total coverage across all samples was chosen to infer the taxonomic distributions and facilitate across-sample comparisons (Table S2, SCG frequency). The abundance of functional genes was obtained using “anvi-estimate-metabolism”, which utilizes the annotated KOs in the contigs-database and calculates the coverage of each KO as a proxy for abundance. To account for varying sequencing depth across samples, we normalized the coverage of the KOs using the coverage of previously selected SCG. To minimize batch effects in metagenomic data, we used the “frequency” method of the “isContaminant” function in the Decontam R package [58], which identifies potential contaminants by exploiting the frequency of each feature in relation to the input DNA concentration. The algorithm relies on a statistical model to identify contaminated features in metagenomic data based on the assumption that the frequencies of contaminated features are inversely proportional to input DNA concentration. Overall, 33 features out of 6300 in our KO tables (Table S4) were identified.

### Genome-resolved metagenomics

To facilitate the binning algorithms with differential coverage signals, we conducted read recruitment using BWA MEM (v0.7.17) [56]. Read files of the same sampling site were independently mapped to the contigs of the corresponding site, resulting in six SAM files per sample, for a total of 108 files. We converted the resulting SAM files into BAM formats using samtools (v1.13), and sorted the BAM files using “anvi-init-bam”. We then profiled each BAM file using “anvi-profile” and combined profile databases of each sample using “anvi-merge”, which resulted in 6 × 3 = 18 merged profile databases. We exported the coverage information stored in the merged profile databases using “anvi-export-splits-and-coverages”. Binning was conducted using CONCOCT(v1.1.0) [59], Maxbin2(v2.2.7) [60], and Metabat2(v2.15) [61], and integrated using DASTool (v1.1.3) [62]. Bins were manually refined using “anvi-refine” based on the tetranucleotide frequency of the contigs within each bin, differential coverage signals of contigs across samples at the same sampling site, and taxonomic information assigned to the contigs. We retained bins with greater than 50% completeness and less than 10% redundancy based on SCGs, and referred to them as metagenome-assembled genomes (MAGs). The program dRep (v3.3.0) was used to calculate average nucleotide identity between MAGs among sites and form clustering dendrograms [63]. Taxonomic classification of the MAGs was conducted using GTDB-tk (v2.1.0) against GTDB (release 207v2) [50, 51]. An explanation of why two GTDB releases were used was provide in supplementary text 4. We annotated the MAGs using “anvi-estimate-metabolism”. The phylogenomic tree was constructed with GToTree v1.7.06 [64], using the prepackaged single-copy gene-set for all domains of life (16 target genes) [65]. For the construction of the phylogenomic tree, we incorporated one representative genome of each Order in GTDB using the “gtt-subset-GTDB-accessions” functions of GToTree, which included 1521 genomes, along with the 230 MAGs recovered in our study. Genomes containing less than 50% of the target single-copy genes were dropped upon phylogenomic reconstruction by setting “-G” to 0.5, resulting in a total of 1368 representative genomes and 159 MAGs utilized in the final tree.

### Taxonomic, phylogenetic, and gene neighborhood analyses

To retrieve HgcAB sequences, we imported HMMs of HgcAB from the Hg-MATE-Db (v1.01142021) [32] using “anvi-run-hmm” into Anvi’o contigs databases with *E*-values of 1 × 10^−50^ and 1 × 10^−30^, respectively [66]. We exported the HMM hits of HgcA from the contigs databases using “anvi-get-sequences-for-hmm-hits”. A multiple sequence alignment of retrieved HgcA sequences was created using MAFFT (v7.471; --auto) [67]. To eliminate paralogs of HgcA, we removed the sequences without the conserved putative cap helix motif [N(V/I)WCA(A/G)GK] reported previously [6]. We further filtered the sequences by retaining only sequences with more than four transmembrane domains as identified by TMHMM (v.2.0) [68]. See Table S3 for details regarding the number of sequences retained through each of these steps. We classified HgcA taxonomy following a previously described method [69]. Briefly, the query sequences were placed on a pre-built reference HgcA phylogenetic tree and taxonomy was assigned based on each queried branch’s lowest common ancestor [69]. We obtained the coverage value of each HgcA sequence stored in contigs databases according to their gene caller ID using “anvi-export-gene-coverage-and-detection”. We used single-copy gene (SCG)-normalized sequence coverage of HgcA as a proxy for the absolute abundance of Hg methylators (i.e., absolute HgcA abundance = unnormalized HgcA coverage/SCG coverage), to account for variations in sequencing depth. The relative abundance of HgcA belonging to specific taxa was determined by dividing the taxon-specific abundance by the total absolute abundance, and then multiplying by 100 to express the result as a percentage (i.e., taxon-specific relative abundance = taxon-specific absolute abundance/total absolute abundance × 100). We dereplicated the HgcA sequences belonging to the same site using CD-HIT (v4.8.1) [70] with a 99% sequence identity cutoff. A maximum-likelihood phylogenetic tree of resulting HgcA sequences was constructed using PhyML (v3.3.2) [71] with 1000 bootstrap replicates. The evolutionary model, LG + Γ_4_, was selected according to the Bayesian Information Criterion using the phangorn package (2.7.0) in R [72]. Tree multifurcations were resolved using the “multi2di” function in the phangorn package. Phylogenetic clustering analysis of the HgcA was subsequently conducted using BaTS [73]. For this, 1000 bootstrapped phylogenetic trees of *rpoB*, *glnA*, and *merA* amino acid sequences were obtained by first creating ten individual 100 bootstrapped alignments using seqboot from the phylip package (v3.698) [74] with distinct random number seeds. The 10 × 100 bootstrapped alignments were analyzed with fasttree (v2.1.11) [75] to build bootstrapped phylogenetic trees, which were then concatenated and fed into BaTS for phylogenetic clustering analysis.

To identify genes located next to *hgcAB* in our metagenomic assemblies and genomes of confirmed Hg methylators, we used `anvi-export-locus`. We retained contigs that have both *hgcAB* and at least one more gene. Out of a total of 5.78 million contigs greater than 1 kb across the metagenomic assemblies, 511 contigs met the above criteria (~0.009%). Taxonomic classification of the *hgcAB* + contigs was conducted using Kaiju (v1.9) [76] against the NCBI RefSeq (release 210) [77] and `anvi-import-taxonomy-for-genes` for integration of the classification results into Anvi’o contigs databases.

### Ordination and statistical analyses

We performed principal component analysis (PCA) using the prcomp function in the Vegan (2.5-7) R package [78] on geochemical measurements and standardized the variance within geochemical measurements using the “scale” argument before conducting PCA. We performed principal coordinate analysis of the whole microbial community and Hg methylators community at the phylum level with Bray–Curtis dissimilarity using the Phyloseq package [79]. The percentage relative abundance of the microbial community was used for constructing the dissimilarity matrix for beta diversity analyses. Differential abundance analysis of the functional genes was conducted using the differential gene expression analysis based on the negative binomial distribution (DESeq2) method [80] Briefly, we compared the unnormalized abundance of the functional genes from the three sites to obtain log_2_ fold changes (LFC) and Benjamini–Hotchberg (BH) adjusted *P* values with a significance threshold of 0.01. Pairwise comparisons between sites were conducted using the ‘contrast’ function in the DESeq2 R package. A positive LFC for a comparison of a gene between site A and site B indicates that the gene in site A is more abundant than in site B, and vice versa. The distance-based redundancy analysis (dbRDA) examining the relationship between Hg methylator community compositions and geochemical variables was conducted using the capscale() function in the Vegan (2.5–7) package [78]. The model was constructed based on a Bray-Curtis dissimilarity matrix calculated from the relative abundance of the Hg methylators across the samples after square-root transformation and Wisconsin double standardization (i.e., *capscale(formula =* *species* ~ *THg* + *MeHg* + *MeHg/THg + Chloride + Nitrate + Sulfate* + *pH* + *EC* + *TDS, data = environment, distance* = *"bray", sqrt.dist* = *TRUE, metaMDSdist* = *TRUE)*). The overall significance of the test and the significance of individual explanatory variables were assessed with permutation using the anova() function. Spearman’s rank correlation coefficients (*ρ*) and the corresponding *P*-value were calculated using the rcorr() function of the Hmisc package (4.7-2) [81] in R.

## Results and discussion

### Archaea as potential key players in Hg transformations

We profiled metagenomic data at the contigs level to infer microbial diversity and abundance in rice paddies using a SCG, *rpS11*, coding for the prokaryotic ribosomal protein S11, as it was the most abundant SCG across the samples. The three sites sampled were abbreviated as HX, GX, and SK, ordered by increasing THg concentrations.

Across all three sites, *Proteobacteria* and *Desulfobacterota* were prevalent, occupying 18.6–35.9% and 8.0–28.5% of the communities, respectively (Fig. 1b, Table S2). Other major bacterial taxa found at HX and GX include *Chloroflexota, Acidobacteriota, Eisenbacteria, and Actinobacteriota*, each representing over 5% of the microbial abundance. At GX, we observed a unique enrichment of *Nitrospirota* (mean abundance: 7.84 ± 1.31%, pairwise Wilcoxon test, FDR corrected *P* < 0.005, Table S5). Importantly, our data revealed significant archaeal presence at SK (pairwise Wilcoxon test, FDR corrected *P* < 0.05, Table S5), accounting for on average 21.68 ± 3.91% of the relative abundance and is primarily constituted by *Halobacteriota* (mean abundance: 9.58 ± 1.05%), *Thermoplasmatota* (mean abundance: 6.08 ± 1.18 %) and *Thermoproteota* (mean abundance: 5.14 ± 2.87%),whereas on average less than 5% of the microbial communities at HX and GX were of archaeal origin (Table S2). An increase in archaeal relative abundance at SK was associated with a significant drop in the abundance of several other phyla compared to HX and GX, including *Eisenbacteria*, *Gemmatimonadota*, *Methylomirabilota* and *Nitrospirota* (pairwise Wilcoxon test, FDR corrected *P* < 0.05, Table S2). A positive correlation between Hg content and Archaeal abundance, is to be taken carefully as that it might be context-dependent, with the observed archaeal enrichment resulting from differences in Hg speciation (i.e., the nature and abundance of ligands Hg is bound to), among other variables in these environments. Other heavy metals (i.e., Cd, Cr, Cu, As, and Pb) did not covary with Hg concentrations (see Table S2 in Pu et al. [13] for details on these heavy metals).

Through metagenomic binning and refinement, we generated 35 high-quality MAGs (>90% completeness, <10% redundancy) and 195 medium-quality MAGs (>50% completeness, <10% redundancy) (Table S6). MAGs identified at HX, GX, and SK were recovered from 12, 8, and, 14 phylum-level lineages according to GTDB classifications (Fig. 1e; Table. S5). Mirroring that in the contigs-level taxonomy, 35% of MAGs recovered at SK are classified to archaeal phyla, whereas archaeal MAGs only constitute around 4% and 13% at HX and GX, respectively. We assessed genome novelty using taxonomies assigned by GTDB-tk [50] based on concatenated protein reference trees and average nucleotide identity (ANI) [63]. Nearly all MAGs were unclassified at the species level (97%), while 12% and 3% of the MAGs were unclassified at the genus and family level, respectively (Table S7). Our analyses thus expanded the genomic archive of microbes found in mining-impacted rice paddies. Based on ANI [63], genomes recovered from different sites share no similarities at the strain level (>99% ANI), whereas only one common species (>95% ANI) was shared between GX and SK (*Myxococcota FEN-1143*, see cluster 17_1 in Fig. S1). The lack of genome similarity among paddy communities across the three sites, together with the observation that microbial community structures were distinctive according to the geochemical heterogeneity in the soil (Figs. 1c, d, S2) indicate an environmental filtering effect on the microbes by the unique geochemical conditions of the paddy systems; Hg concentration may be one of such geochemical variables.

### Methanogens likely prevail under severe Hg concentrations

**Fig. 2:**
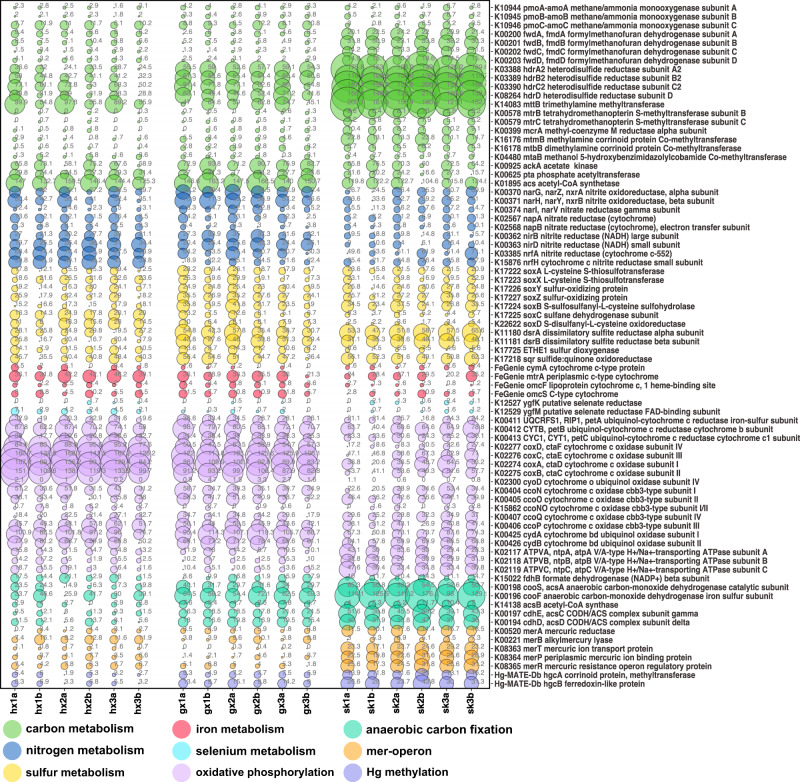
Normalized abundance of functional genes related to various metabolic pathways. (Abundance is normalized by dividing the coverage of the gene by the coverage of the most abundant single-copy core genes, *rpS11*, across samples.) Feature names are shown on the right side of the Y-axis. Features with their name starting with K are genes retrieved from the KEGG ortholog database based on HMMs. The iron-cycling genes starting with FeGenie were recovered using HMMs from the FeGenie [110] database. The *hgcAB* genes were recovered using HMMs built previously [32]. The values inside the bubble represent the normalized coverage values. Bubble sizes reflect normalized abundance of each feature in a way that maximize the across-sample contrast (Bubble sizes were determined by subtracting the coverage value of each feature in each row from the smallest coverage value at the same row).

We characterized the abundance of functional genes participating mostly in catabolic pathways, at the contigs level, relevant to carbon, sulfur and nitrogen associated compounds, as well as oxidative phosphorylation and Hg-cycling genes (Fig. 2), to understand site-specific microbial metabolism and physiology.

Notably, we observed evidence of a thriving methanogenic community at highly-contaminated SK, demonstrated by a significantly greater relative abundance of genes associated with methanogenesis (*mcrA*, *mttB*, *fwdABCD*, *hdrABC;* BH adjusted *P* < 0.01, LFC < 0, Table S8, supplementary Text 3). We found that *Methanomicrobiales*, a hydrogenotrophic methanogen [82], were present across all sites but represented a non-negligible proportion of the microbial community at SK (6.6%; Table S6), which is in accordance with the abundance of *hdrABC* (Fig. 2), coding the heterodisulfide reductase specifically present in hydrogenotrophic methanogens [83]. Furthermore, SK exhibited significantly more *mttB* gene (encoding for trimethylamine methyltransferase, K14083) compared to the other sites, suggesting that methylotrophic methanogens that utilize trimethylamine as a substrate for methanogenesis were abundant. However, the low abundance of *mtmB* (monomethylamine methyltransferases, K16176), *mtbB1* (dimethylamine methyltransferases, K16178), and *mtaB* (methanol:MtaC protein Co-methyltransferase, K04480; Table S4) implies that monomethylamine, dimethylamine and methanol were not major substrates for methylotrophic methanogens at SK [84]. Genes associated with acetoclastic methanogenesis were almost uniformly abundant across sites, including *ackA* (acetate kinase, K00925), *pta* (phosphate acetyltransferase, K00625), and *acs* (acetyl-CoA synthetase, K01895), indicating that acetoclastic methanogens were universally present in paddy soil regardless of Hg content. Our previous work conducted using paddy soil at the abandoned Hg mining site showed that the addition of methanogenesis inhibitor significantly increased MeHg production and decreased demethylation, suggesting that methanogens played a major role in MeHg degradation as THg concentration ([THg]) increased [24]. These observations were attributed to increased 1) oxidative demethylation performed by methanogens and 2) competition of methanogens for substrates with other Hg methylating guilds, as Hg contamination increased [24]. Here, combined with genomic evidence presented in this study, it is likely that methanogens associated with MeHg degradation or competition with other Hg-methylating methanogens under high Hg concentrations are hydrogenotrophic or methylotrophic, although further investigations are warranted. Critically, a previous study showed that acetoclastic methanogens might be associated with MeHg degradation in alder swamps, a net sink for MeHg [85]. These speculated substrate-dependence of important MeHg degradation pathways (here *via* trimethylamine-dependent methanogenesis) may offer tractable insights (i.e., by adjusting the concentration of the substrate) into managing MeHg contamination in Hg-impacted systems. Importantly, our findings implied that SK could be a potential site for exploring the metabolic pathways involved in oxidative demethylation, possibly by using Hg and carbon isotope fractionation to find a joint isotope signature in potential enzymes [86], and then using transcriptomics to detect changes in the expression of specific genes in response to MeHg degradation assays under various exposure conditions.

### Increasing dominance of archaeal Hg methylators at high Hg concentrations

**Fig. 3:**
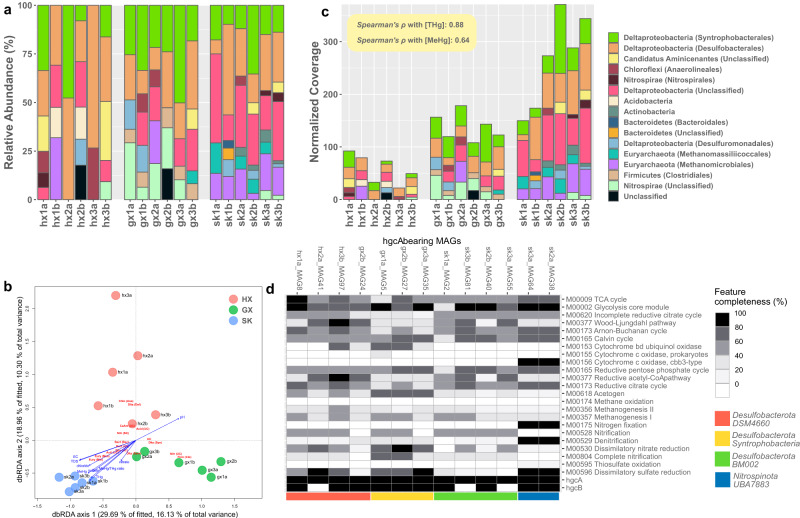
Taxonomic and functional overview of Hg methylators. **a** Taxonomic classification of the *hgcA* sequences at the phylum level and their abundance in relative abundance. **b** A dbRDA investigating the relationship between *hgcA*-based community composition Hg methylators and the geochemical parameters in our dataset of 18 samples from the three sampling sites. (Abbreviations: Phylum-level: Acid: *Acidobacteria*, Acti: *Actinobacteria*, Bact: *Bacteroidetes*, CaAM: *Candidatus Aminicenantes*, Chlo: *Chloroflexi*, Dlta: *Deltaproteobacteria*, Eury *Euryarchaeota*, UC unclassified, Class-level: Ana: *Anaerolineales*, Bac: *Bacteroidales*, Clo: *Clostridiales*, Def: *Desulfobacterales*, Dem: *Desulfuromonadales*, Mei: *Methanomicrobiales*, Mes: *Methanomassiliicoccales*) **c** Taxonomic classification of the *hgcA* sequences at the phylum level and their abundance in normalized gene coverage. **d** Metabolic profile showing the completeness of essential metabolic pathways in MAGs of putative Hg methylators recovered in this study. The colored bars at the bottom represent their corresponding taxonomic classification at the phylum and class level.

Identifying the microbial players involved in Hg methylation under various Hg concentrations is crucial to understanding the controlling factors on environmental MeHg dynamics. Phylogenetic analysis based on *hgcA* showed that Hg methylators belong to ten distinct phyla (NCBI taxonomy). *Syntrophobacterales* and *Desulfobacterales* order of *Deltaproteobacteria* dominated nearly all samples (Fig. 3a), suggesting that fermentative and sulfate-reducing Hg methylators are prevalent across paddy soils regardless of [THg] and [SO_4_^2-^] (Table S1). We noticed that unclassified *Nitrospirae* (i.e., *Nitrospirota*) are virtually exclusively present at GX, occupying 14.2% of the read coverages on average. At the family level, *Desulfuromonadales*-like *hgcA* were all classified as *Geobacteraceae* (Table S9), a group of iron(III)-reducing bacteria that methylates Hg in diverse environments including rice paddies, and mercury-affected freshwater sediments [16, 87–89]. We show that the relative abundance of *Geobacteraceae*-like *hgcA* comprises, on average, only a very small proportion (<5%) of all *hgcA* sequences and was not uniformly present across sites. This finding corroborates a previous experimental result showing that iron amendment in the form of FeOOH at SK seldom affected Hg methylation rate [24]. Therefore, evidence suggests that iron-reducing Hg methylators might only play a minor role in MeHg production in these contaminated rice paddies.

Hg methylators of *Euryarchaeota* phylum occupied, on average, 5.3%, 4.8%, and 18.9% of Hg methylators abundance at HX, GX, and SK, respectively (Fig. 3a). Specifically, *Methanomicrobiales*-associated Hg methylators, an order within *Euryarchaeota* that performs hydrogenotrophic methanogenesis [90], were ubiquitously present at SK (13.0% of the relative abundance), which is in line with *Methanomicrobiales*-associated *rpS11* gene occupying a substantial proportion SCG coverage (Table S6) and significantly greater abundance of *hdrABC* genes (Fig. 2) at the abandoned mining site. Overall, the ordination plot (Fig. 3b) based on the Bray-Curtis dissimilarity shows the mining-impacted sites (i.e., GX and SK) tended to cluster together in terms of the taxonomic composition of Hg methylators. In contrast, taxonomic composition of Hg methylators from the control site are more dispersed. Here, we show that 1) the taxonomic distribution of Hg methylators (Fig. 3b) does not follow that of the whole microbial community (Fig. 1d), 2) the taxonomic distribution of Hg methylators tend to be more homogenized in paddy soil as [THg] increases, and 3) archaeal Hg methylators become increasingly dominant as [THg] increased. Again, the implications of the above observations should be considered under the constraints that THg might not be the only geochemical variable affecting community structure.

### *hgcAB* abundance positively correlates with both [THg] and [MeHg]

The ordination analysis (dbRDA) showed that 54.35% of the total variance in the taxonomic composition of Hg methylators was explained by the measured geochemical variables (Fig. 3b). Permutation test indicates that THg was the only geochemical variable that exhibited a significant effect in shaping the composition of Hg methylators (*F* = 2.3215, *P* = 0.006), although the constraints are not overall significant (*F* = 1.0581, *p* = 0.331). We conducted Spearman’s ranked correlation analysis to examine the correlation between *hgcAB* abundance and [THg], as well as with [MeHg] in our samples. *hgcAB* abundance exhibited strong positive correlations with both [THg] (*hgcA*: *ρ* = 0.88, *P* = 2.2 × 10^−16^; *hgcB*: *ρ* = 0.89, *P* = 2.2 × 10^−16^) and [MeHg] (*hgcA*: *ρ* = 0.64, *P* = 0.0055; *hgcB*: *ρ* = 0.68, *P* = 0.0023) across the sites (Figs. 2, 3c). While some studies found that the abundance of *hgcA* poorly correlated with [THg] and [MeHg] [26, 27], only one study reported that in rice paddies a greater abundance of *hgcAB* was associated with increasing [THg] and [MeHg] [17], and another found that *Geobacter*-associated *hgcA* positively correlated with [MeHg] [16]. These discrepancies between *hgcAB* abundance and environmental Hg content could possibly be attributed to several methodological limitations. For instance, the qPCR-based approaches [17, 33] are prone to primer bias, resulting in a preferential recovery of *hgc*A from *Deltaproteobacteria* [91]. Furthermore, metagenomic studies implementing HMM could underestimate the diversity of Hg methylators [16] when the HMM was built with a limited number of sequences [69]. Finally, the unstandardized gene abundance estimation method could lead to controversial results [92].

Although not impartial, our approach to estimating *hgcA* abundance is likely less biased because we extracted full-length HgcA from contigs using an HMM built from an improved HgcA database with more than a thousand known sequences [32]. We recovered HgcA with a recently published consensus protocol [31] and used gene coverage as a proxy for abundance. However, due to a relatively small sample size here (*n* = 18), the results may not be generalizable to other environments, even with an improved methodology. Considering the constitutive expression of *hgcAB* [93], and the dramatically different methylation rates among Hg methylators [8], elucidating the controlling factors of [MeHg] in the environment still requires a multifaceted approach combining investigations on transcript and protein abundances, enzymatic capacities of methylation and demethylation proteins (i.e., HgcAB and MerB) associated with different genotypes, as well as Hg bioavailability [27, 87, 94].

### Enhanced demethylation potential points to alternative mechanisms of Hg resistance

MeHg demethylation represents a non-negligible reaction contributing to limit net MeHg accumulation in the environment. As such, we also examined the occurrence of *merB*, coding the alkylmercury lyase (MerB). The abundance of *merB* was significantly greater at HX, the control site, than at SK, the most contaminated site (BH adjusted *P* = 3.54 × 10^−4^, LFC = 1.21 Fig. 2, S3). Additionally, *merB* at HX was also more abundant than at GX, indicated by a positive LFC, (BH adjusted *P* = 0.014, LFC = 0.41 Fig. 2, S3), although without statistical significance, which might be a result of an insufficient sample size. Consistent with our genomic results showing an excessive occurrence of *merB* at HX, a previous study showed that HX exhibited the highest microbial degradation of MeHg among the three sites [24], indicating that, in rice paddies with lower [THg], MerB-facilitating aerobic reductive demethylation might be the dominant MeHg degradation pathway.

We show that at HX, the normalized abundance of *merB* is, in general, greater than that of *merA* (Fig. 2), suggesting that *merB* at the control site might be expressed independently of the *mer*-operon, a phenomenon that has been suggested previously [95]. The activity of MerB alone could pose considerable cellular cytotoxicity to microbes due to the lack of subsequent Hg(II) reduction typically conducted by the mercuric reductase, MerA [95]. Therefore, it is plausible that a *merB:merA* > 1 suggests the existence of alternative Hg(II) reduction processes, unrelated to the *mer*-operon. Such process may involve fermentative Hg(II) reduction [96] or anaerobic metal reduction [97], which warrants further investigation. Reliance on *merB-*only coupled to co-metabolic pathways to remove Hg(II) from the cells, may prove less costly for the cell. Here, our results imply the presence of demethylating microbes that lack *merA*, making HX an ideal site to screen for such organisms, which we intend to do.

### *Nitrospinota UBA7883*—a novel facultative anaerobic mercury methylator

The metabolic potential of *hgcA*-bearing MAGs was investigated by annotating them using KEGG modules (Fig. 3d, Table S10). Nearly all *hgcA*-bearing MAGs demonstrated complete or partial pathways of dissimilatory nitrate reduction (M00530), dissimilatory sulfate reductions (M00596), glycolysis (M00002), and TCA cycle (M00009; Fig. 3d), indicating an anaerobic chemoheterotrophic lifestyle, possibly due to selection of these anaerobes from the community as a result of the prevalence of electron acceptors (e.g., nitrate, sulfate; Table S1) and carbon sources. All *Syntrophobacteria* and some *DSM4660* (*Desulfatiglans anilini*) Hg methylators carried genes encoding the cytochrome bd ubiquinol oxidase (M00153), potentially allowing them to survive in sub-oxic environments [98]. Notably, another study recovered *hgc* + MAGs belonging to Marinimicrobia and encoding several terminal oxygenases from suboxic ocean water, further supporting the potential oxygen tolerance of some Hg methylators [9].

We observed two novel *hgcA*-bearing MAGs (sk3a_MAG64, sk2a_MAG38) associated with *UBA7883* class of *Nitrospinota* in the abandoned mining site SK. *Nitrospinota-*like (or *Nitrospina*) *hgcA* have been previously identified primarily in oceanic and microaerophilic settings, including the Antarctic Sea ice [99], equatorial North pacific [10], mesopelagic zone of the East China sea [100] and subsurface water of the global ocean [9, 101]. To our knowledge, this is the first time potential *Nitrospinota* Hg methylators have been discovered in rice paddies. These MAGs showed exceptional completeness (at >97%) and contamination scores (<3%; Table S6). Taxonomic classification only resolved to the order level, indicate the lack of representative genomes in public databases and the novelty of these microbes. The *UBA7883* MAGs encode complete or nearly complete dissimilatory sulfate reduction pathways (M00596), denitrification pathways (M00529), and a terminal oxidase, cytochrome c oxidase cbb3-type (M00156; Fig. 3d, Table S10), making them a potential facultative anaerobe. Various catabolic pathways indicates that *UBA7883* can switch between energy generation strategies under a wide range of redox potentials utilizing sulfate, nitrite, and oxygen as terminal electron acceptors, enabling them to survive in the fluctuating water levels characterizing paddy systems. Curiously, the complete nitrogen fixation pathway (M00175) is also detected in *UBA7883* MAGs. Given the lack of the nodulation module (M00664), these putative nitrogen-fixers may represent non-symbiotic diazotrophs, which benefit from the anaerobic nature of flooded paddy soil (i.e., as anoxic environments could protect the nitrogenase of the nitrogen-fixation pathway from oxygen toxicity), and contribute significantly to the organic nitrogen pool in rice paddies [102]. Additionally, Hg methylators encoding nitrogen-fixing ability have been suspected to be important contributors to MeHg accumulation in sediments impacted by acid mine drainage, potentially because such an ability increased their competitiveness under nitrogen-limiting conditions [103]. Here, the symbiotic status of the putative nitrogen-fixing Hg methylators, and whether they feed crops with ammonia and MeHg simultaneously, remain to be answered.

### Did *hgcA* evolve under the selective constraint of Hg?

One outstanding question pertains to the role of Hg methylation. Hg methylation could be 1) a way to purposefully limit the accumulation of intracellular Hg(II), as the methylation of Hg(II) is possibly coupled with the export of MeHg out of the cell [36], or 2) a co-metabolic process occurring accidentally [34, 35]. A recent study, based on an evolutionary analysis of *hgc* genes, suggested that Hg methylation may have provided microbes with a competitive advantage in the primitive ocean, with MeHg acting as an antimicrobial compound [37].

In the context of our study, we assessed if *hgcA* exhibited divergent genotypes under various [THg], as a change in gene sequences could lead to altered protein structure, potentially influencing methylation rate, a sign that THg might have influenced *hgcA* evolution. Accordingly, we predicted that should [THg] influence the genotypic makeups of *hgcA*, we would observe phylogenetic clustering of *hgcA* according to THg content. Such clustering pattern would not be expected to exist for housekeeping genes such as *rpoB*, because *rpoB* phylogenies should not be directly affected by Hg pressure [38, 104]. For this, we employed the Bayesian Tip-association Significance testing (BaTS) [73] on bootstrapped maximum likelihood (ML) phylogenetic trees constructed with translated *hgcA*, *merA*, and two housekeeping genes, *glnA* (encoding glutamine synthetase) and *rpoB* (encoding β-subunit of the bacterial RNA polymerase) across the three sites (Table 1). BaTS tests the null hypothesis that sites are associated randomly with the phylogenetic tips using a Bayesian MCMC approach [73]. We found that all tested genes exhibited significant clustering by geographic locations (i.e., sampling site), with *P* < 0.001 for the association index (AI) and the parsimony score (PS). However, the result is not congruent with our prediction, because all genes exhibited phylogenetic clustering, including the housekeeping genes. Our observation could be explained by a strong environmental filtering effect present across the sites that structured the microbial community and the genetic makeup of genes, independent of Hg. Useful insights into the role of Hg on the evolution of *hgcA* could be gleaned from a reductionist approach relying on experimental evolution conducted in the lab.

**Table 1:** Phylogeny-trait analysis results for *hgcA*, *merA*, *rpoB* and *glnA*.

Statistic	Observed mean	Null mean	*P* value (BaTS null hypothesis test)
	(95% HPD CIs)	(95% HPD CIs)	
*hgcA*			
AI	2.79 (2.17–3.44)	11.48 (10.07–12.90)	<0.001
PS	40.53 (39–42)	68.87 (64.15–72.76)	<0.001
MC (Huaxi)	3.0 (3–4)	1.79 (1.03–2.90)	0.02
MC (Gouxi)	5.09 (5–6)	2.76 (2.00–4)	0.008
MC (Sikeng)	12.23 (9–18)	4.80 (3.31–6.94)	0.002
*merA*			
AI	1.98 (1.56–2.36)	7.37 (6.25–8.44)	<0.001
PS	20.21 (19–22)	42.96 (39.63–46.01)	<0.001
MC (Huaxi)	7.33 (5–8)	2 (1.17–3)	0.001
MC (Gouxi)	3.44 (3–4)	2.16 (1.35–3.00)	0.125
MC (Sikeng)	39.57 (35–43)	4.12 (3.01–5.92)	0.001
*rpoB*			
AI	13.49 (12.03–15.01)	48.39 (45.55–51.18)	<0.001
PS	157.35 (153–162)	308.44 (297.64–318.10)	<0.001
MC (Huaxi)	15.00 (15–15)	5.15 (3.88–7.01)	0.001
MC (Gouxi)	7.32 (5–8)	3.28 (2.38–4.45)	0.002
MC (Sikeng)	10.87 (9–11)	3.33 (2.53–4.77))	0.001
*glnA*			
AI	62.24 (58.38–66.01)	182.49 (177.44–187.44)	<0.001
PS	658.63 (645–672)	1229.89 (1209.61–1249.89)	<0.001
MC (Huaxi)	24.90 (25–26)	5.63 (4.57–7.11)	0.001
MC (Gouxi)	10.21 (9–13)	4.80 (4.01–6.03)	0.001
MC (Sikeng)	21.52 (13–31)	4.06 (3.24–5.14)	0.001

*AI* Association index, *PS* Parsimony score, *MC* Monophyletic clade, *HDP Cis* highest posterior density confidence intervals.

### A putative *hgcAB* regulator in Archaeal Hg methylators

**Fig. 4:**
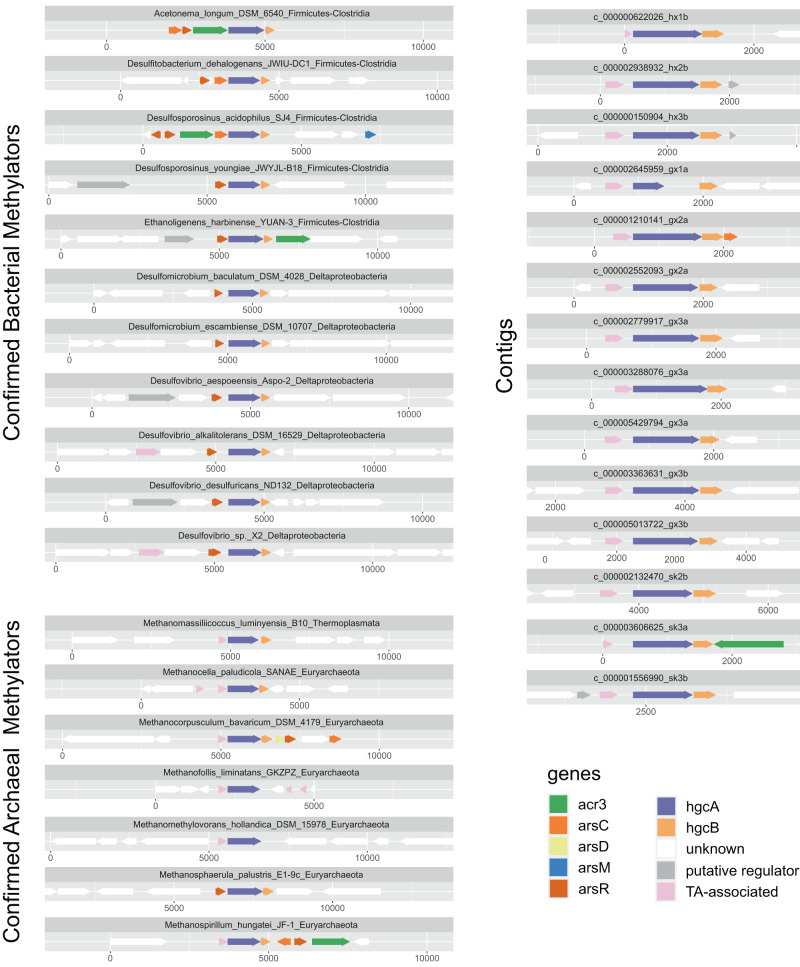
Gene neighborhoods of selected confirmed Hg methylators illustrating the association of arsenic-related genes and the TA-gene with *hgcAB* in bacteria and archaea, respectively (see Fig. S4 for the full list of *hgcAB* gene neighborhoods in confirmed Hg methylators). The same association between the TA-gene and *hgcAB* was also demonstrated in selective contigs recovered in our metagenomic assemblies across the sampling sites. On top of each gene neighborhood plot, strain and phylum were indicated for the confirmed Hg methylators, and the contig numbers were specified.

The variables controlling the expression of *hgcAB* are important insights into the environmental controls of Hg methylation. Recently, the role of the microbial arsenical resistance operon, particularly the *arsR* gene that codes a transcriptional repressor [105], was hypothesized to be coupled to the expression of *hgcAB*. Subsequent genomic studies investigating synteny at *hgcA* loci of various methylating strains found a co-transcribed putative *arsR* gene upstream of *hgcA* [39], as well as several other *ars*-associated genes (e.g., *acr3/arsB*, *arsM*, *arsC*) located near *hgcAB* in genomes of Hg methylators [66, 106]. Notably, the transcriptional regulation of *hgcAB* by an ArsR-like regulator (Pfam PF01022) and the arsenic-induced transcriptional changes of *hgcAB* was later experimentally confirmed in *Pseudodesulfovibrio mercurii* ND132, implying a close association of arsenic metabolism with Hg methylation [107]. To evaluate whether methylating genes recovered here show comparable genomic signatures in association with arsenic metabolism, we analyzed contigs containing both *hgcAB* and at least two additional cooccurring genes. Our result shows that out of all contigs (i.e., 35) that met the criteria, four contigs contain at least one *ars*-associated gene such as *arsC*, *acr3*/*arsB*, *arsA*, and *arsD* (Fig. 4, Table S9). However, we failed to retrieve *arsR* in any selected contigs.

Critically, we noticed that 14 *hgcAB*-bearing contigs (i.e., ~39% of all contigs) have a gene associated with the microbial toxin-antitoxin (TA) system directly upstream of *hgcA* (Fig. 4). Annotation of the TA gene based on different databases returned inconsistent results, from which the Clusters of Orthologous Genes (COGs) identified it as the antitoxin component (*prlF*) of the YhaV-PrlF toxin-antitoxin module and *abrB*, coding a bifunctional DNA-binding transcriptional regulator (COG2002) (Table S11). The gene was also identified as *prlF* (antitoxin PrlF, K19156) and *mazE* (antitoxin MazE, PF04014.21) by KEGG and Pfam, respectively, although with less convincing e-values (Table S11). Taxonomic classification revealed that 50% of the TA-related *hgcAB+* contigs have an archaeal origin, all belonging to the *Methanomicrobia* class (Table S12). Conversely, only a small proportion (i.e., ~10%;) of the non-TA encoding *hgcAB*+ contigs were identified as archaeal (Table S12).

To further examine whether different regulators of Hg methylation likely exist between bacteria and archaea, we analyzed 31 genomes of experimentally confirmed Hg methylators obtained from axenic cultures. We created neighborhood gene plots to demonstrate the relatedness among genes (Table S11). We observed that 14 genomes have the putative *arsR* gene upstream of *hgcA* within three ORFs (Figs. 4, S4), from which 13 (or 92.8%) are bacterial genomes, whereas only one archaeal genome, resolved to genus *Methanosphaerula*, contains *arsR*-like gene upstream of *hgcA*. In contrast, eight genomes have the TA-associated *prlF* gene directly upstream of *hgcA*, and 6 (or 75%) are archaeal, consistent with that found in the contig-level data showing archaeal Hg methylators tend to have the TA gene alongside *hgcA*. Note that some bacterial Hg methylators have putative regulators upstream of *hgcA* that are neither associated with the *ars*-operon nor the TA system. For example, we identified genes coding a transcriptional regulator of the GntR family (COG2188) on *Desulfitobacterium metallireducens* 853-15A, a transcriptional regulator of the MarR family (COG184) on *Dethiobacter alkaliphilus* AHT1, and *Geobacter daltonii* FRC-32.

Here, the TA-associated gene was identified as *prlF* and *mazE*, homologous genes encoding antitoxins of the type II TA system, typically organized as operons [108, 109]. Expression of type II TA operons is generally autoregulated at the transcriptional level by the toxin-antitoxin complex to maintain a homeostatic state within the cell [109], and it is plausible that *hgcAB* might be co-regulated with the TA operon in archaea. The finding of the TA gene also raises an important question as to whether bacteria and archaea have incorporated *hgcAB* into different regulatory systems. Further investigation into whether these putative transcriptional regulators are co-transcribed with *hgcAB* and their controlling factors might offer new insights into the evolution and expression of *hgcAB* in archaea, and ultimately, the biochemical constraints of Hg methylation.

## Supplementary information

Supplementary Information

Supplementary MATERIAL Table S1

Supplementary Table S2

Supplementary Table S3

Supplementary Table S4

Supplementary Table S5

Supplementary Table S6

Supplementary Table S7

Supplementary Table S8

Supplementary Table S9

Supplementary Table S10

Supplementary Table S11

Supplementary Table S12

Supplementary Table S13

## Data Availability

Raw metagenomic sequences were submitted to the NCBI SRA under the following accession numbers: SRR15313068, SRR15313069, SRR15313070, SRR15313071, SRR15313072, SRR15313073. The MAGs and bioinformatic pipeline used in this project have been deposited in GitHub (https://github.com/rzhan186/gy2020_bioinformatics).
