# Supplementary material for: Mining-impacted rice paddies select for Archaeal methylators and reveal a putative (Archaeal) regulator of mercury methylation
Source: ISME Commun. 2023 Jul 15;3:74. doi: 10.1038/s43705-023-00277-x (PMC10349881; doi:10.1038/s43705-023-00277-x)
Supplement: Supplementary file 1 — Supplementary Information [file 43705_2023_277_MOESM1_ESM.pdf]

Supplementary Information

Mining-impacted rice paddies select for Archaeal methylators and reveal a putative (Archaeal) regulator of mercury methylation

Rui Zhang[1], Stéphane Aris-Brosou[1,2] Veronika Storck[3], Jiang Liu[4], Mahmoud A. Abdelhafiz [4,5], Xinbin Feng[4], Bo Meng[4], Alexandre J. Poulain[1]

[1] Department of Biology, University of Ottawa, Ottawa, ON K1N 6N5, Canada

[2] Department of Mathematics and Statistics, University of Ottawa, Ottawa, ON K1N 6N5, Canada

[3] Department of Civil Engineering, Polytechnique Montréal, Montréal, QC H3C 3A7, Canada

[4] State Key Laboratory of Environmental Geochemistry, Institute of Geochemistry, Chinese Academy of Sciences, Guiyang 550081, China

[5] University of Chinese Academy of Sciences, Beijing 100049, China

Supplementary Text 1: [THg] span several orders of magnitude across the sampling sites while [MeHg] remains relatively constant

Total mercury concentrations ([THg]) varied across the three sites due to unique Hg exposure histories, ranging from 0.23 mg/kg to more than 400 mg/kg. However, despite an exponential increase in [THg] across the three sites (i.e., 0.21−513 mg kg−1 dry wt. soil), MeHg concentrations ([MeHg]) on average only increased by about three-fold. Regardless, SK showed significantly higher [MeHg] than both HX and GX, respectively (pairwise Wilcoxon test, FDR corrected $p < 0.05$), but no significant differences in [MeHg] were found between soil samples taken at HX and GX (pairwise Wilcoxon test, FDR corrected $p < 0.005$). We found that the paddy soil [MeHg] measured in this study is not in accordance with a previous study conducted in rice paddies along the same contamination gradient in 2017 (1), where the highest [MeHg] was detected at GX. The inconsistency in [MeHg] is potentially caused by a change in sampling location and demonstrates the spatial and temporal variability of [MeHg] in rice paddies despite a consistent [THg] signature. However, we observed a reverse relationship between [THg] and MeHg to THg ratio (MeHg/THg) among the three sites, consistent with the previous study in contaminated rice paddies (1) and aquatic systems (2). In addition, to better understand the variability of [THg] and [MeHg] at the three sites, we took measurements along their soil profiles, extending 25 cm below the surface (Fig. S2). While [THg] tends to be relatively stable along the soil profiles, [MeHg] at GX and SK peaked at the soil-water interface, reaching concentrations up to 9.25 ng/g and 8.42 ng/g, respectively, then rapidly dropped and stabilized down the profiles (Fig. S2), a phenomenon that has been observed in freshwater sediments (2,3). Overall, the principal component analysis (PCA) based on standardized geochemical measurements of the soil samples (Fig. 1c) shows that HX and GX share similar characteristics despite being geographically distant

(Fig. 1a). In contrast, SK showed distinct geochemical properties in the ordination space from the other two sites (Fig. 1c).

Supplementary Text 2: Sample preparation, geochemical analyses and sequencing

Upon disturbing the paddy water, the oxidation-reduction potential (ORP), conductivity, dissolved oxygen (DO) (Shanghai San-Xin instrumentation, Inc. Model number: SX751, China), and pH (Leici E-201F pH probe, China) were measured at each site (Table S1). Three 10 m by 10 m plots were subsequently established randomly at each site. Within each plot, We chose three spots randomly with paddy water coverage to collect the surface soil (1-5 cm below the surface) and the rhizosphere soil (10-20 cm below the surface). Surface soil samples were collected using a sterilized shovel. Rhizosphere soil samples were collected by pulling out the rice plants and cutting a piece of the root where the soil was attached using sterilized scissors. Samples were subsequently transferred into whirl-paks and placed in a cooler filled with ice packs. Liquid nitrogen was poured into the cooler intermittently to keep the temperature down. Samples were held in the cooler for no longer than 12 hours before transferring to a freezer for storage at -20 °C. As a final note, we decided to not treat surface soil and rhizosphere samples as separate groups in the following analyses due to our suspicion that the true rhizosphere might not have been captured because of difficulties in removing attached paddy soil from the roots on site. Therefore, all samples were treated equally as paddy soils. Sampling techniques of soil pore water and soil core was described in an early publication (4). Analysis of soil Hg content and pore water geochemistry were conducted following previously described methods (1,5).

Frozen soil samples were first thawed at room temperature. To account for the variability in microbiomes posed by the heterogeneous nature of the soil environment, we subsequently created composite samples of surface soil and rhizosphere soil respectively, by pooling each type of sample from the same plot under sterile conditions, resulting in a total of 18 composite samples out of the 48 raw samples. Next, 25 ml of 0.5% saline solution was poured into the rhizosphere composite samples and vortexed for 10 minutes to remove the

attached soil from the plant roots. Upon DNA extraction, the composite samples were centrifuged for 10 minutes at 4000 rpm using a Kaida® TD5A centrifuge, and the supernatants were removed. The soil DNA was extracted using the DNeasy® Powersoil® Kit following the manufacturer's instructions. DNA purity and concentration were validated using a Qubit fluorometer at sequencing companies (Table S13). Overall, DNA extraction was completed within four days after sampling, and a total of 19 DNA samples were obtained (18 composite soil samples + 1 control). Shotgun metagenomic sequencing of the samples was conducted at LC-Bio (Hangzhou, China) on an Illumina NovaSeq 6000 platform (paired-end 150), with a read depth of ~30 Gbp per sample (Table S13). To validate the results of the former company, one sample plus the control was sent to Genewiz (Suzhou, China) for sequencing using the same sequencing platform and settings. To minimize batch effects (6) in our sequencing data, we took extra caution during the DNA extraction process where all samples were performed using the same extraction kit by the same personnel. DNA library preparation was also subsequently completed by the same personnel with identical reagents.

Supplementary Text 3: Contigs level microbial functional profiles

First, genes coding the subunits of several oxidase systems of the prokaryotic electron transport chain were more abundantly present in HX and GX metagenomes compared to that in SK, including (1) *UQCRFS1*, *CTYB* (coding the cytochrome bc1 complex, a.k.a. the cytochrome c reductase, KEGG module: M00152), (2) *cydA* (cytochrome bd ubiquinol oxidase, M00153), (3) *coxA*, *coxB*, *coxC*, *coxD* (cytochrome c oxidase aa_3 type, M00155), (4) *ccoN*, *ccoP* (cytochrome c oxidase cbb_3 type, M00156) (BH adjusted $P < 0.01$, LFC > 0 , Table S8).

These terminal oxidases play essential roles in cellular energetics and are potential indicators of the underlying environmental conditions. For example, the expression of cytochrome c oxidase cbb_3 type, a bacteria-specific oxidase, and cytochrome bd ubiquinol oxidase are favored under oxic and hypoxic conditions due to their high affinity for oxygen(7,8). Conversely, cytochrome o ubiquinol oxidase (M00417) preferentially functions in oxygen-rich environments (9). Additionally, electron transfer capacity of the cytochrome bc1 complex is stimulated by elevated oxygen concentrations (10). However, gene abundance might be a biased predictor of protein expression and activity. Notably, *cyoD* was the only annotated gene coding for a subunit of the cytochrome o ubiquinol oxidase across the metagenomes, together with its little coverages, suggesting that none of the sites have experienced oxygen-sufficient conditions. Overall, the enrichment of various terminal oxidase systems implies that microbial communities inhabiting HX and GX were subjected to labile oxygen concentration and have managed to adapt to the alteration in redox potential through the expression of various oxidases (11). In the contrary, the relative scarcity of the oxidases at SK suggests that the site had maintained a lower redox potentially stably that sustained minimal aerobic respiration.

Since flooded rice paddies are mostly anaerobic systems that are also significant sources of methane (12), it is plausible that methanotrophy dominated by anaerobic methane oxidation (AOM) is prevalent. As expected, we identified genes involved in AOM (*mcrAB*, *mtrC*, *frhB*) across the sampling sites, with a significantly higher abundance at SK, indicating a thriving community of anaerobic methane-oxidizing archaea (13). However, our statistical approach identified genes coding the methane monooxygenase subunits (*pmoABC*) as contaminating features, impeding our inference on aerobic methanotrophy, which also explains the lack of an apparent trend across the three sites. From an anabolic perspective, SK is characterized by a higher abundance of genes coding components involved in the reductive acetyl-CoA pathway (a.k.a. the Wood-Ljungdahl (WL) pathway, M00377), including the subunits the carbon monoxide dehydrogenase (*cooS*, *cooF*, *cdhE*, *cdhD*) and the acetyl-CoA synthase (*acsB*). The WL pathway is essential for carbon fixation, such as the biosynthesis of acetate using H_2 and CO_2 as electron donors and acceptors, respectively, and is indicative of obligate anaerobes due to the oxygen sensitivity of various enzymes involved in this pathway (14).

In addition to carbon compounds, the surveyed paddy soils were rich in terminal electron acceptors such as nitrate and sulfate. Despite a greater average nitrate concentration at SK (3.78 ± 1.21 mg/l, n=6), in comparison to that of HX (2.54 ± 0.52 mg/l) and GX (3.10 ± 2.13 mg/l), several genes involved in dissimilatory nitrate reduction (i.e., *narG*, *narH*, *nirB*, *nirD*) were more enriched at HX and GX (BH adjusted $P < 0.01$, $LFC > 0$ Table S8). The microbial energetics associated with the cycling of sulfur-related compounds can be coupled to either their reduction or oxidation. Across the sampled soils, a significant difference in sulfate concentration ($[SO_4^{2-}]$) was observed between GX (150.80 ± 47.89 mg/l, n=6) and SK (1095.52 ± 381.20 mg/l, n=6). Although GX demonstrated smaller abundance of *dsrAB* ($LFC < 0$), the differences are not statistically discernable (BH adjusted $P > 0.01$, Table S8),

implying a comparable potential of dissimilatory sulfate reduction, a catabolic reaction using sulfate as the TEA. In contrast, HX showed the lowest abundance of *dsrAB* compared to the other two sites (BH adjusted P<0.01, LFC < 0, Fig. 2, S3), although the [SO_4^{2-}] is similar to that of GX. Interestingly, regardless of the discrepancies in [SO_4^{2-}] and *dsrAB* abundance, the pore water of soil samples from all sites displayed relatively consistent sulfide concentration ([S^{2-}]), a byproduct of dissimilatory sulfate reduction (Table. 1). It is plausible that the activities of sulfate-reducing bacteria (SRB), which can be lithotrophic or organotrophic, are limited by electron donors such as H_2 and organic compounds. However, in-situ measurements of H_2 and organics are required to validate this assumption. The differences in abundance of metabolic genes related to dissimilatory nitrate reduction and dissimilatory sulfate reduction imply that the options of electron acceptors for microbial community at SK tend to be limited to chemical compounds with lower redox potential, such as SO_4^-/HS^-. Alternatively, it is likely that the choices for electron acceptors are more diverse at HX and GX, as manifested by the higher relative coverage of genes related to aerobic and anaerobic respirations, suggesting a more frequent fluctuation of redox potential.

HX and GX exhibited a greater abundance of the various subunits of the prokaryotic NADH quinone oxidoreductase (except for the subunit F) (Table S2). The enzyme is broadly present in bacteria with diverse energetic strategies, but rarely in archaea (15), coinciding with our observation of the bacterial dominance at HX and GX. In contrast, SK exhibited a larger abundance of genes coding the V/A-type H+/Na+-transporting ATPase (Table S2), which functions as an ATP synthase but is mainly present in archaea and some extremophilic bacteria (16), in agreement with the observation that SK had a higher proportion of archaeal ribosomal protein encoding genes.

Lastly, the bacterial mer-operon typically encodes several enzymes that function collectively to confer microbes the resistance to Hg(II), including a cytoplasmic mercuric reductase (MerA) responsible for Hg(II) to Hg(0) conversion, a periplasmic Hg(II) sequester protein (MerP), a membrane-bound transporter protein (MerT), and a metal-responsive regulator (MerR) (17). Our results showed significant enrichment of *merATPR* at SK compared to the other sites (BH adjusted $P < 0.01$, $LFC < 0$, Table S8). The greater abundance of *merA* at SK reflects an enhanced potential of Hg(II) reduction and hence detoxification, possibly stimulated by an elevated bioavailable Hg(II). Moreover, we noticed that average [THg] at GX was over a hundred times higher than that at HX. Yet, there was no statistically significant difference in *merA* abundance between the two sites.

Supplementary Text 4: The use of two versions of GTDB in this study

We implemented two versions of the GTDB (202 and 207) (18) for taxonomic profiling at the contigs and MAGs level, respectively. GTDB ver. 202 was published on April 7, 2021, and ver. 207 was released a year later on April 8, 2022. The GTDB releases are indexed on RefSeq, and each new release is accompanied by some updates on species representatives (i.e., representative genomes). GTDB ver. 202 spans 258,406 genomes organized into 47,894 species clusters (https://gtdb.ecogenomic.org/stats/r202), while ver. 207 spans 317,542 genomes organized into 65,703 species clusters (https://gtdb.ecogenomic.org/stats/r207). Although the number of representative genomes has increased dramatically, the number of phyla only increased from 146 to 166.

Here, ver. 202 was mainly used to describe the taxonomic composition in our assembled contigs at the phylum level (Fig. 1b) using the Anvi'o software, and the reason we used release 202 is that it was the most up-to-date version at the time we were conducting the analysis. On the other hand, release 207 was used to describe the taxonomies of the MAGs, and such information was used beyond the phyla level. However, it is unlikely that the use of different GTDB versions results in differences in interpretations, because no change in phylum names have been made between the two releases, thus we expect that the results remain unchanged should we had used ver. 207 in Anvi'o for taxonomic profiling.

Reference

1. Wu Q, Hu H, Meng B, Wang B, Poulain AJ, Zhang H, et al. Methanogenesis Is an Important Process in Controlling MeHg Concentration in Rice Paddy Soils Affected by Mining Activities. Environ Sci Technol. 2020 Nov 3;54(21):13517–26.

2. Bravo AG, Cosio C, Amouroux D, Zopfi J, Chevalley PA, Spangenberg JE, et al. Extremely elevated methyl mercury levels in water, sediment and organisms in a Romanian reservoir affected by release of mercury from a chlor-alkali plant. Water Research. 2014 Feb;49:391–405.

3. Gilmour CC, Henry EA, Mitchell R. Sulfate stimulation of mercury methylation in freshwater sediments. Environ Sci Technol. 1992 Nov;26(11):2281–7.

4. Zhao L, Anderson CWN, Qiu G, Meng B, Wang D, Feng X. Mercury methylation in paddy soil: source and distribution of mercury species at a Hg mining area, Guizhou Province, China. Biogeosciences. 2016 Apr 27;13(8):2429–40.

5. Liu J, Lu B, Poulain AJ, Zhang R, Zhang T, Feng X, et al. The underappreciated role of natural organic matter bond Hg(II) and nanoparticulate HgS as substrates for methylation in paddy soils across a Hg concentration gradient. Environmental Pollution. 2022 Jan;292:118321.

6. Leek JT, Scharpf RB, Bravo HC, Simcha D, Langmead B, Johnson WE, et al. Tackling the widespread and critical impact of batch effects in high-throughput data. Nat Rev Genet. 2010 Oct;11(10):733–9.

7. Borisov VB, Gennis RB, Hemp J, Verkhovsky MI. The cytochrome bd respiratory oxygen reductases. Biochimica et Biophysica Acta (BBA) - Bioenergetics. 2011 Nov 1;1807(11):1398–413.

8. Pitcher RS, Watmough NJ. The bacterial cytochrome cbb3 oxidases. Biochimica et Biophysica Acta (BBA) - Bioenergetics. 2004 Apr 12;1655:388–99.

9. Cotter PA, Chepuri V, Gennis RB, Gunsalus RP. Cytochrome o (cyoABCDE) and d (cydAB) oxidase gene expression in Escherichia coli is regulated by oxygen, pH, and the fnr gene product. J Bacteriol. 1990 Nov;172(11):6333–8.

10. Zhou F, Yin Y, Su T, Yu L, Yu CA. Oxygen dependent electron transfer in the cytochrome bc1 complex. Biochimica et Biophysica Acta (BBA) - Bioenergetics. 2012 Dec 1;1817(12):2103–9.

11. Lin H, Ascher DB, Myung Y, Lamborg CH, Hallam SJ, Gionfriddo CM, et al. Mercury methylation by metabolically versatile and cosmopolitan marine bacteria. ISME J [Internet]. 2021 Jan 27 [cited 2021 Mar 14]; Available from: http://www.nature.com/articles/s41396-020-00889-4

12. Neue HU. Methane Emission from Rice Fields. BioScience. 1993;43(7):466–74.

13. Magnuson E, Altshuler I, Fernández-Martínez MÁ, Chen YJ, Maggiori C, Goordial J, et al. Active lithoautotrophic and methane-oxidizing microbial community in an anoxic, sub-zero, and hypersaline High Arctic spring. ISME J [Internet]. 2022 Apr 8 [cited 2022 Apr 27]; Available from: https://www.nature.com/articles/s41396-022-01233-8

14. Ragsdale SW, Pierce E. Acetogenesis and the Wood-Ljungdahl Pathway of CO2 Fixation. Biochim Biophys Acta. 2008 Dec;1784(12):1873–98.

15. Spero MA, Aylward FO, Currie CR, Donohue TJ. Phylogenomic Analysis and Predicted Physiological Role of the Proton-Translocating NADH:Quinone Oxidoreductase (Complex I) Across Bacteria. Harwood CS, editor. mBio [Internet]. 2015 May [cited 2021 Dec 22];6(2). Available from: https://journals.asm.org/doi/10.1128/mBio.00389-15

16. Kühlbrandt W, Davies KM. Rotary ATPases: A New Twist to an Ancient Machine. Trends in Biochemical Sciences. 2016 Jan 1;41(1):106–16.

17. Barkay T, Miller SM, Summers AO. Bacterial mercury resistance from atoms to ecosystems. FEMS Microbiol Rev. 2003 Jun;27(2–3):355–84.

18. Parks DH, Chuvochina M, Chaumeil PA, Rinke C, Mussig AJ, Hugenholtz P. A complete domain-to-species taxonomy for Bacteria and Archaea. Nat Biotechnol. 2020 Sep;38(9):1079–86.

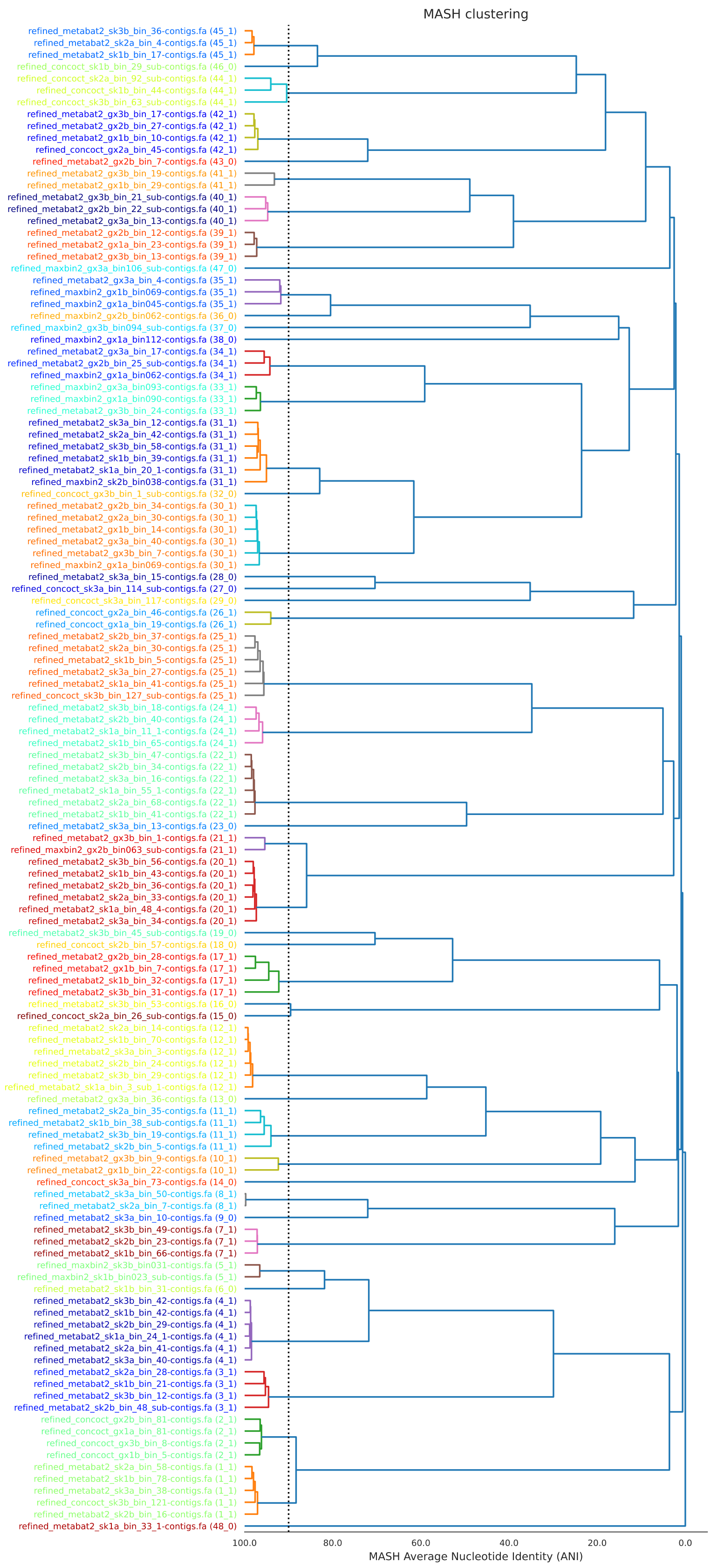

Figure S1. A clustering dendrogram showing the pair-wise Mash distance between MAGs recovered at GX and SK. The dotted line is drawn at 90% ANI.

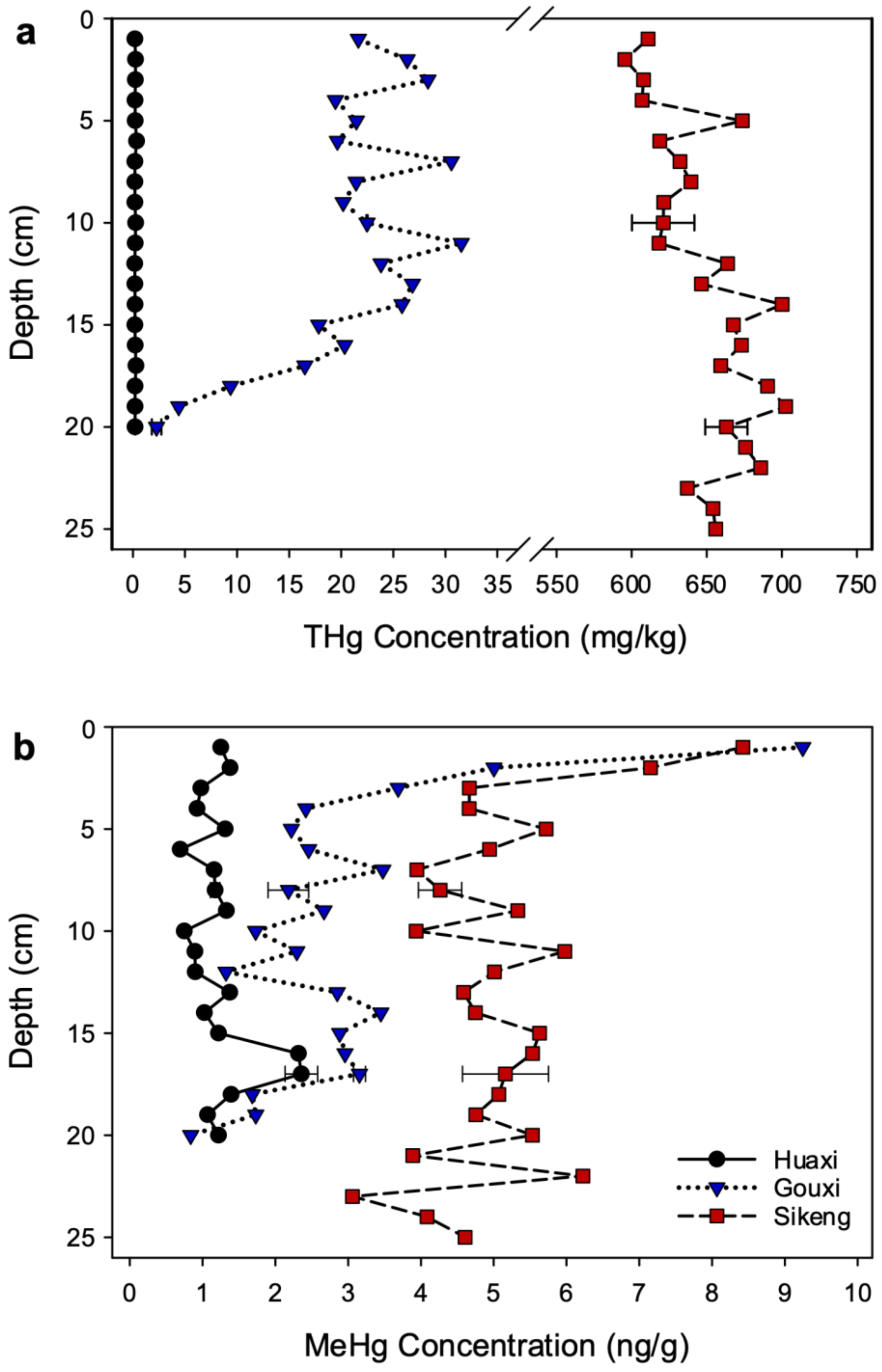

Figure S2. Soil profile of **a.** total Hg and **b.** MeHg concentrations at the three sites. (Error bars represent standard deviations as samples at certain depths were measured multiple times.

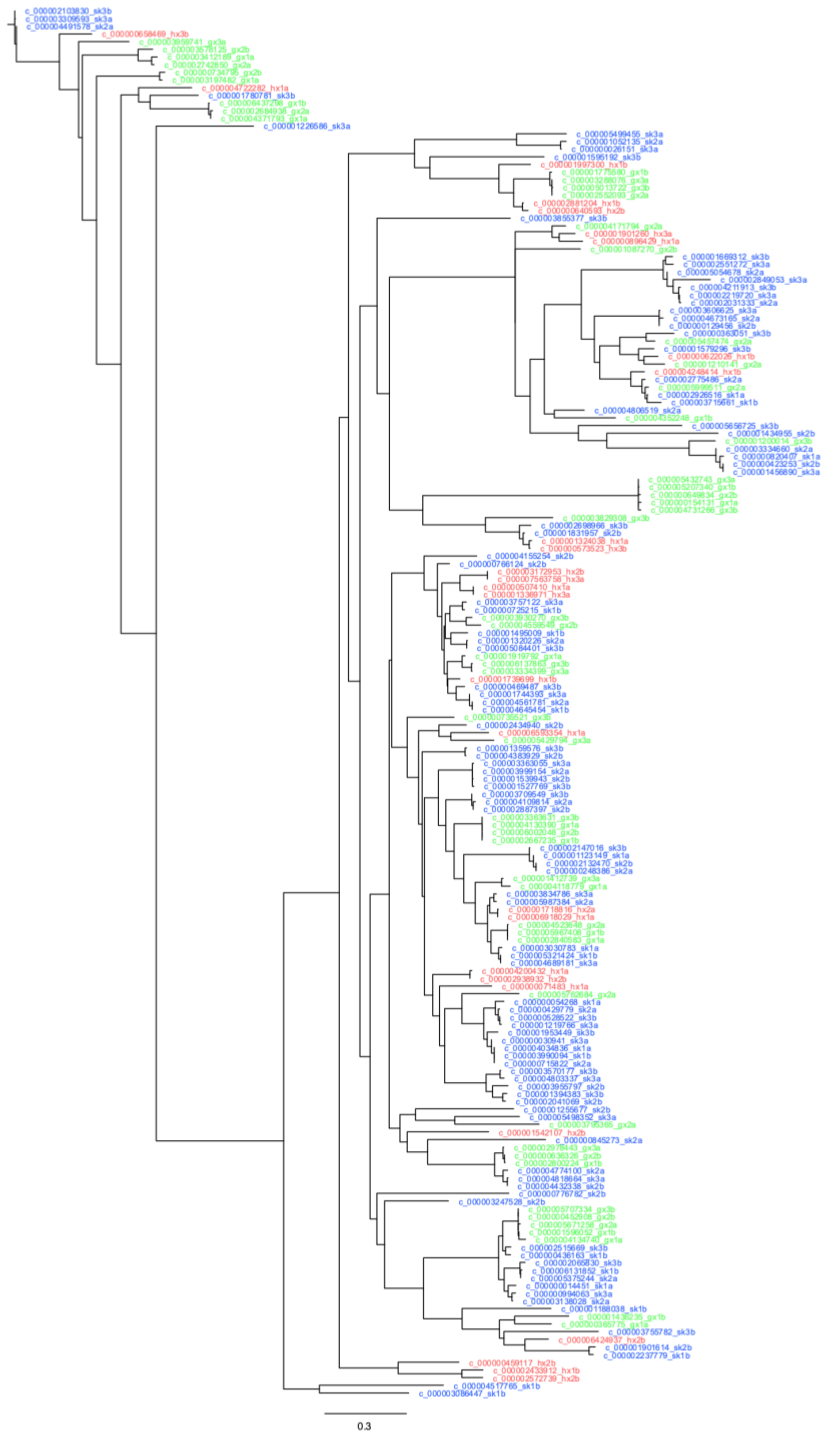

Figure S3. Maximum likelihood phylogenetic tree of HgcA amino acid sequences. HgcA sequences recovered from contigs are highlighted according to sites (Red: Huaxi; Green: Gouxi; Blue: Sikeng). Branch names reflect the contig number where the gene was recovered. The tree was constructed using PhyML with 1000 bootstrap replicates based on an LG model with a Gamma rate of heterogeneity with 4 discrete categories (-m LG -f e -c 4 -a e). The evolutionary model was selected using the Phangorn R package according to AIC. Tree visualization was conducted using FigTree.

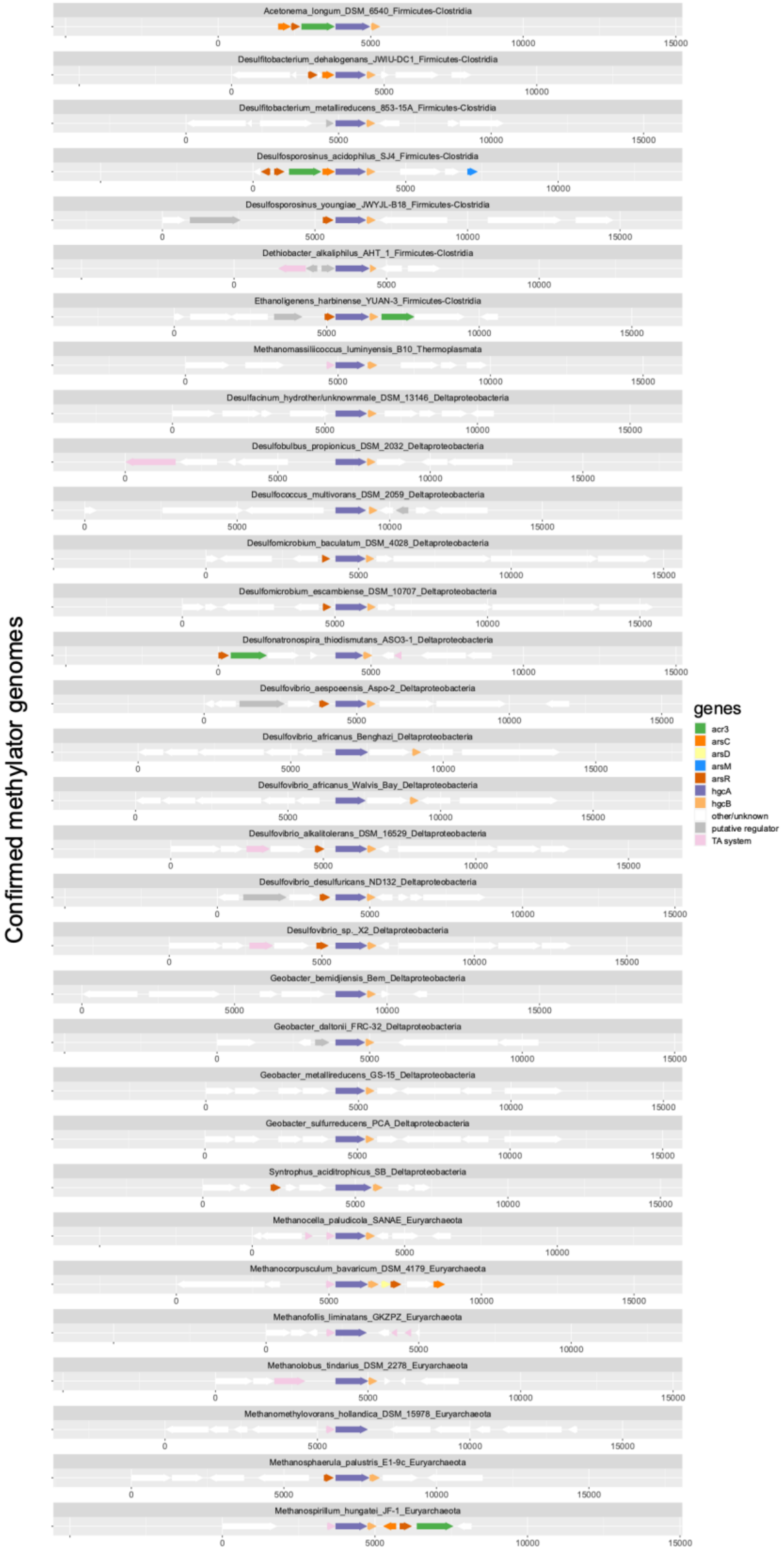

Figure S4. Gene neighborhoods of all examined confirmed zxHg methylators, demonstrating the association of the TA gene with *hgcAB*.

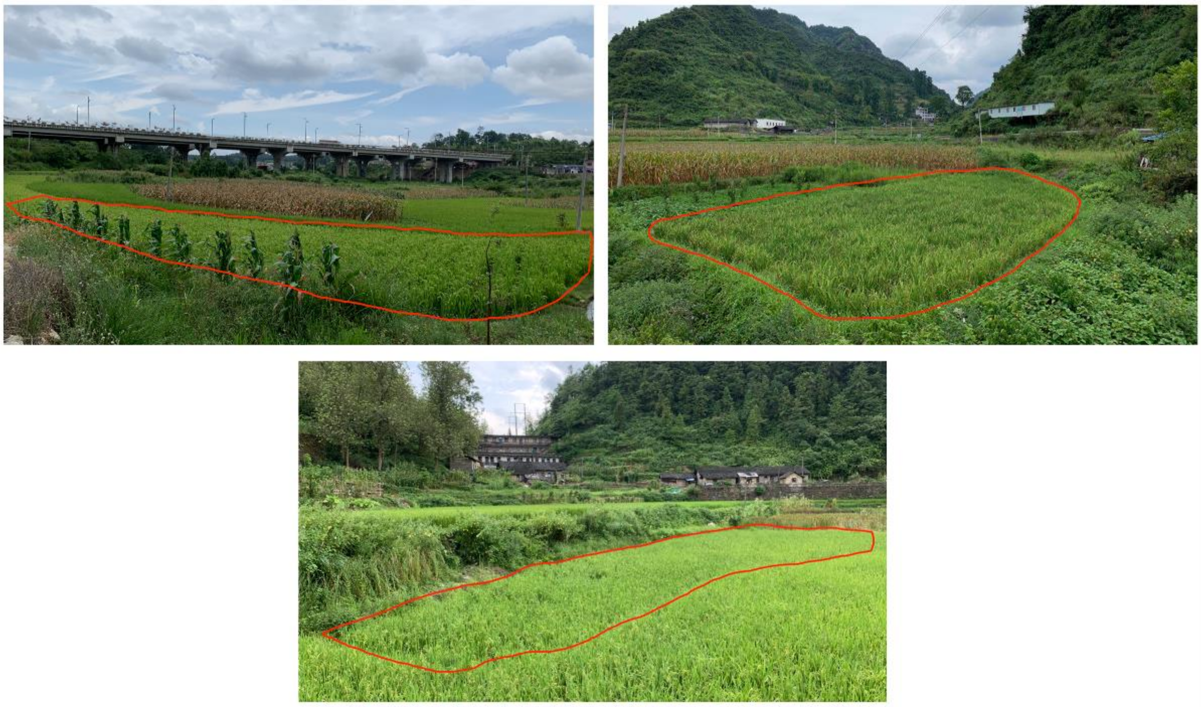

Figure S5. Pictures of the sampling sites. Samples were collected randomly within the circled area (top left: Huaxi; top right: Gouxi; bottom: Sikeng).
